# Facial and Periorbital Cellulitis due to Skin Peeling with Jet Stream by an Unauthorized Person

**DOI:** 10.1155/2014/529153

**Published:** 2014-04-16

**Authors:** Asli Feride Kaptanoglu, Didem Mullaaziz, Kaya Suer

**Affiliations:** ^1^Department of Dermatology, Near East University Hospital, Lefkosa, North Cyprus, Mersin 10, Turkey; ^2^Department of Infectious Diseases and Clinical Microbiology, Near East University Hospital, Lefkosa, North Cyprus, Mersin 10, Turkey

## Abstract

Technologies and devices for cosmetic procedures are developing with each passing day. However, increased and unauthorized use of such emerging technologies may also lead to increases in unexpected results and complications as well. Here, we report a case of facial cellulitis after a “beauty parlor” session of skin cleaning with jet stream peeling device in 19-year old female patient for the first time. Complications due to improper and unauthorized use of jet stream peeling devices may also cause doubts about the safety and impair the reputation of the technology as well. In order to avoid irreversible complications, local authorities should follow the technology and update the regulations where the dermatologists should take an active role.

## 1. Introduction

Nowadays, cosmetic applications and medical devices that promise to improve the skin problems are popular and widely used and some of them are easily accessed due to the lack of government regulations in some countries. Peeling skin with a jet stream (shortly named as jet-peeling) is a new technology for cosmetic resurfacing and deep cleansing of the skin. It is reported as a safe and effective tool for the usual indications for facial peeling such as resurfacing, wrinkles, scars, and acne treatment [1]. Indications of jet-peeling are also developing, such as delivering antiseptic and anesthetic solutions to the deeper layers of skin [2]. However, increased and unauthorized use of such emerging technologies may also lead to increases in unexpected results and complications as well. Infectious complications after cosmetic procedures by unauthorized persons are commonly reported [3–5]. To the best of our literature search, facial cellulitis developing after a session of jet-peeling has not been reported previously. The aim of reporting this case is to draw attention to the possible complications which might be caused by the misuse of cosmetic devices.

## 2. Case Report

**Figure 1:**
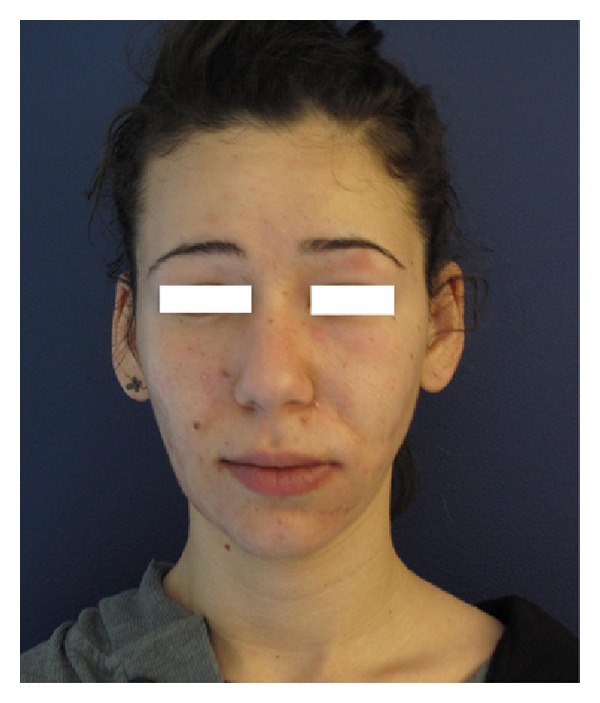
Erythema, edema, and pustules on the left side of the face.

Nineteen-year-old previously healthy female patient was admitted with swelling, pain, and redness on the left side of her face and left eye region. Her admittance to our clinic was the first time of medical admittance. The patient declared the presence of only blackheads (comedones) until two days ago. However, she had a history of jet-peeling session in “*a beauty parlor*” for the facial skin cleansing and peeling 5 days ago. She had no other disease and was using neither a systemic nor a topical treatment for acne and did not use any medication for any other reason. She had no history of previous dentistry treatment. Two days prior to her admission, left side of her face got edematous and followed by increasing redness, swelling, and pain which spread to her eye and neck. Physical examination revealed unilateral diffuse edema including periorbital region, erythema, pustules, and cysts with a purulent discharge on the left side of the face (Figure 1). Palpation was painful and there were lymphadenopathies in the anterior cervical and submandibular chains. She had mild fever as 38.1 C. Laboratory tests showed elevated C Reactive Protein (CRP: 2.39 mg/dL) and white blood cells (12.900/uL). Her blood glucose, IgE levels, Blood Urea Nitrogen (BUN), creatinine, and liver tests were in normal ranges. A direct smear and microbiological culture were performed from the purulent discharge. In the microscopic evaluation with gram stain polymorphonuclear leukocytes, gram (−) bacillus and gram (+) coccus were seen. Bacterial culture with blood agar revealed* Staphylococcus epidermidis* reproduction. In the antibiogram there was resistance to only clindamycin, penicillin, and erythromycin. She was diagnosed as facial-periorbital cellulitis and antibiotherapy was planned. She was initially given cefazolin and continued after the culture as the antibiogram revealed sensitivity to cefazolin. After 10 days of antibiotic regimen, her facial edema, erythema, and purulent lesions were recovered, with a residue of comedones and slightly erythematous papules of acne.

## 3. Discussion

Cellulitis is an infection of the deep dermis and subcutaneous tissue that manifest as areas of erythema, swelling, warmth, and tenderness. Bacteria typically gain access to the dermis via a break in the skin barrier in immunocompetent persons. Cellulitis in immunocompetent adults is mostly due to Streptococcus* spp.* and* Staph aureus* [6].* Staphylococcus* is common colonizers of skin. Particularly,* Staphylococcus epidermidis* is a commensal bacterium of the human skin and regarded as an innocuous commensal microorganism. However, there are many reports regarding it as an “accidental pathogen” responsible for the nosocomial infections in immunocompromised or medical prosthesis inserted patients [7, 8]. In our patient, insertion of bacteria might not be with surgery, but with a strong jet stream pressure which even can enhance drug delivery from intact skin [2]. We were not the initial operator of the device. Hence, neither we nor the patient had information about the technique, depth of peel, number of passes, anestesia, or the type of the fluid. The patient reported that she had no knowledge if her skin has been well disinfected before the treatment.

Jet-peel seems to be a noninvasive, effective, and easy-to-apply facial peeling method and such “innocent” properties provide an attractive option for beauty salons. The success of a treatment modality in facial resurfacing is based on patient selection as well as ability to manage the side effects even in most developed devices [9]. This unauthorized use and misuse of the device may lead to unexpected and worrisome complications as in our patient. All the techniques which break the epidermal barrier must be performed by a trained practitioner. Jet- peel may not be suitable if the patient has certain medical conditions, such as inflamed or infected skin, possibility of keloid scarring, or pressure urticaria. We strongly emphasize the importance of a good clinical exam before this procedure to avoid the risk of superficial but also deep infection. Also the physician should be aware of the risk of herpes outbreak. In our patient, there was unilateral facial cellulitis indicating an external inoculation of infection. Moreover, as jet-peel has effects on lymphatic drainage, inappropriate and improper use of the device might have caused the spread of the superficial skin infection into deeper skin tissues or the orbita.

## 4. Conclusion

Considering that the data in the literature is few, jet-peeling devices should be used with caution and under the supervision of a dermatologist or plastic surgeon. Whether severe or not, acne should only be treated by medical doctors. Beauticians' dare to treat acne is not only unlawful but also can result in permanent damage to the patients face. Bad complications due to improper use of the device may also cause doubts about the safety and impair the reputation of the technology as well.
